# Nonprofits for Cohesive Cities: Neighborhood Characteristics, Organizational Practices, and their Effects on Social and Systemic Integration

**DOI:** 10.1007/s11266-023-00571-1

**Published:** 2023-05-05

**Authors:** Dominik Karner, Michael Meyer, Lisa Schmidthuber, Daniel Semper, Krystal Laryea

**Affiliations:** 1 Department of Management, WU Vienna University of Economics and Business, Vienna, Austria; 2 School of Management, University of St Andrews, St Andrews, UK; 3 Graduate School of Education, Stanford University, Stanford, USA

**Keywords:** Nonprofit organizations, Urban cohesion, Systemic integration, Neighborhood characteristics, Organizational practices

## Abstract

Nonprofit organizations (NPOs) contribute to vital neighborhoods by building communities of citizens and acting as intermediaries between citizens and organizations. We investigate how NPOs’ engagement in social and systemic integration is shaped by neighborhood characteristics, and how it relates to the organizational practices of managerialism and organizational democracy. We combine survey data with administrative data from a representative sample of NPOs in a major European city. To measure the effect of neighborhood on organizational integration, we separated the city into 7,840 grid cells characterized by population, per capita income, share of immigrant population, and density of organizations. Findings indicate that managerialism positively relates with systemic integration, as organizational democracy relates with social integration. Neighborhood characteristics, however, are not related with NPOs’ engagement in integration. Our findings contribute to research on urban social cohesion by illuminating the interplay between NPOs’ organizing practices, local neighborhoods, and contributions to both forms of integration.

**Supplementary Information:**

The online version contains supplementary material available at 10.1007/s11266-023-00571-1.

## Introduction

Nonprofit organizations (NPOs) contribute to the functioning of cities in manifold ways: they support administrations and communities in meeting societal challenges (Grønbjerg & Paarlberg, 2001). They further urban cohesion by fostering social relations among individuals and between citizens and organizations (McQuarrie & Marwell, 2009), strengthening the social order in urban communities, contributing to city governance, and addressing ecological and societal challenges, such as integration and inequality (Vermeulen et al., 2016a, 2016b).

Extant scholarship has typically considered the effects of social capital on local community development (e.g., Hishida & Shaw, 2014; Hommerich, 2015; Hwang & Young, 2022; Krishna, 2002; Nahapiet & Ghoshal, 1998). Although NPOs have been acknowledged in their capability to produce social capital (e.g., Mutz et al., 2022), the literature has overlooked NPOs’ capacity to *link* citizens with institutional actors beyond the immediate *bonding* within their community and the *bridging* between different communities (Putnam, 2000; Sampson, 1999; Trigilia, 2001). However, as Brandtner and Laryea (2021) argue, NPOs contribute to urban cohesion by engaging in two distinct forms of integration: social and systemic integration. The former refers to the fostering of bonds among citizens, the latter to the intermediation of individuals with institutional resources. Both forms of integration represent cornerstones of urban cohesion as they enhance organizational survival and a resilient social order (Vermeulen et al., 2016a, 2016b; Wang & Vermeulen, 2020).

Despite the richness of scholarship on urban NPOs explaining their prevalence (Bielefeld, 2000; Van Puyvelde & Brown, 2016) and their contributions to social inclusion (e.g., Shier et al., 2022; Vandermeerschen et al., 2017), the full range of systemic integration has not yet been thoroughly considered (Marwell & Morrissey, 2020; Marwell et al., 2020). Particularly our knowledge of ‘how’ and ‘where’ NPOs contribute to both forms of integration remains scarce. We therefore augment Brandtner and Laryea’s (2021) model of NPOs’ engagement in urban integration by analyzing how NPOs’ location and organizational practices relate with integration modes.

For one, location has substantial effects on how NPOs operate, as they often serve constituents in their immediate vicinity and depend on resources provided by local stakeholders (Wolpert, 1993). In consequence, their contributions to urban cohesion vary across neighborhoods (Wu, 2019; Yan et al., 2014), which can reinforce neighborhood disadvantages (Sampson & Graif, 2009).

In addition, NPOs’ engagement in integration is also shaped by organizing practices (Suykens et al., 2022). In European cities, especially in those with a social democratic tradition, NPOs build on a long legacy of democratic forms of organizing based on participatory practices, volunteering and a strong membership base (Maier et al., 2022). However, with NPOs’ organizational practices shifting towards professional management (Hwang & Powell, 2009), NPOs’ capacity to integrate socially is being called into question (Eikenberry & Kluver, 2004).

Distinguishing between managerialist and democratic forms of organizing (Maier & Meyer, 2011), we investigate their effects on NPOs’ engagement in social and systemic integration, and ask: (1) How do urban NPOs engage in social and systemic integration? (2) How does this engagement in social and systemic integration relate to characteristics of the local neighborhoods (per capita income, share of immigrant population, and organization density)? (3) How does this engagement relate to managerialism and organizational democracy? To test these effects, we use a unique dataset of NPOs from the city of Vienna, combining representative survey data of NPOs (*n* = 459) with geographically fine-grained administrative data at the level of the statistical grid unit (250 × 250 m).

Our study contributes in a twofold way. First, by addressing both the social and systemic integration of NPOs in urban contexts, we extend the understanding of the role of NPOs in creating social cohesion and neighborhood vitality beyond the provision of social capital (e.g., Hwang & Young, 2022). We thereby also add to the scholarship on social and systemic integration (Archer, 2007; Brandtner & Laryea, 2021; Esposito, 2020). Second, by zooming into the local context of NPOs, we illuminate the interplay between neighborhoods and NPOs’ contribution to urban cohesion. In addition, our study has also implications for urban policies concerned with promoting urban cohesion, e.g., in urban planning, urban development, and social service provision.

## Theory and Hypotheses

### Nonprofits’ Contributions to Social and Systemic Integration

In their role as drivers of urban cohesion, NPOs provide the space and practices to build social capital (Putnam, 2000) and nurture the development of local communities (Cagney et al., 2020; Klinenberg, 2015; Small & Adler, 2019). “‘Social capital’ refers to features of social organization, such as networks, norms, and trust, that facilitate coordination and cooperation for mutual benefit” (Putnam et al., 1992: 6f), thereby constituting a necessary condition for community development (Nahapiet & Ghoshal, 1998), providing the glue that makes collective action possible (Krishna, 2002). Social capital thus strengthens community identity and its welfare by enabling cooperation and the pursuit of common objectives (Putnam, 1995). For example, Hwang and Young (2022) showed the effects of social capital on local philanthropic engagement. Others highlighted the importance of social capital in disaster recovery by bringing NPOs and volunteers together (Hishida & Shaw, 2014; Kaltenbrunner & Renzl, 2019).

Research has distinguished *bonding* from *bridging* social capital (Putnam, 2000; Woolcock & Narayan, 2000). The first refers to the cohesion of social networks of homogeneous groups of people. This type of social capital enables collective agency, congregational unity, personal connections, and furthers a desire for homogeneity (Leonard & Bellamy, 2015). Especially in poor neighborhoods, close-knit ties allow communities to ‘get by’ (de Souza Briggs, 1998). The second concept–*bridging*–refers to the connections between socially heterogeneous groups, which is argued to help local communities to ‘get ahead’ (Barr, 1998). Therefore, both *bridging* and *bonding* aspects of social capital contribute to social integration of neighborhood communities. However, as Sampson (1999) noted, even if communities exhibit strong interpersonal ties, they might still lack institutional ties. This refers to a third facet of social capital, i.e., the *linking* of people with institutions, which corresponds to systemic integration (Small, 2009; Small et al., 2008).

By creating bonding, bridging, and linking forms of social capital, NPOs are essential drivers of the social and systemic integration of communities. Social integration concerns face-to-face interaction and interpersonal exchange, and relates to membership and volunteering in local NPOs (Ruiz & Ravitch, 2022). In contrast, systemic integration refers to impersonal mechanisms, e.g., bureaucratic rules or money (Marwell and McQuarrie, 2013), which characterize collaboration among organizations. These two concepts draw on Ferdinand Tönnies’ distinction between *Gemeinschaft* and *Gesellschaft* (Tönnies, 1887). Social integration concerns Gemeinschaft, i.e. the private sphere constituted by individual action, whereas systemic integration refers to functional systems such as politics and economy (Bohnen, 1984; Habermas, 1981). Both spheres build an intertwined fabric of institutions typical for modern societies. However, as systemic integration is usually underpinned by power and money, Habermas (1981: 267ff) warns that this may lead to reification and colonialization of the lifeworld.

In urban contexts, the interlocking of *system* and *Lebenswelt* yields a co-evolution of local communities and their organizations (Marquis & Battilana, 2009; Walker & McCarthy, 2010). As spaces are both “socially produced and simultaneously socially producing” (Dale & Burrell, 2008: 6), NPOs and their environment are mutually constitutive: NPOs rely on external resources, while local communities often depend on the services provided by NPOs (Bielefeld, 2000; Wu, 2019). For example, areas with a low level of nonprofit engagement often suffer from low political participation (Fernandez et al., 2022; Hum, 2010), poor community health (Joassart-Marcelli et al., 2011), poor educational outcomes, and high crime rates (Sampson, 2012; Sampson & Graif, 2009). Therefore, neighborhoods exhibit specific needs and resources that determine NPOs’ capacity to integrate socially and/or systemically. Likewise, factors such as age distribution, immigrant population, and wealth may affect the prevalence and activities of NPOs (Grønbjerg & Paarlberg, 2001; Lu, 2017). For example, Gilster et al. (2020) showed that higher levels of neighborhood needs and organizational resources relate to more volunteering. Hence, in poorer neighborhoods NPOs often fulfill specific functions, which are connected to service provision and community building (Katz, 2014; Peck, 2007). We therefore theorize:

#### **H1a**

A low level of average income of citizens in a neighborhood has a positive effect on NPOs’ social integration.

Besides, the share of immigrants is likely to affect NPOs’ engagement in social and systemic integration. Vermeulen et al., (2016a, 2016b) found that a high immigrant population is negatively associated with the density and survival of voluntary leisure organizations. In contrast, the number of specific neighborhood foundations is higher in migrant neighborhoods due to demands of various ethnic groups (Vermeulen et al., 2012), being confronted with specific needs and challenges such as financial pressure, settling and integrating in a new place (Joassart‐Marcelli, 2013; Vermeulen et al., 2012). Apart from informal social networks of friends and family, NPOs are key providers of immigrant services and social integration (Schrover & Vermeulen, 2005). Given that social integration builds upon interpersonal ties based on trust to reduce the social distance within the neighborhood (Hansmann, 1987; Ray & Preston, 2009), we theorize:

#### **H1b**

A higher share immigrant population in the neighborhood has a positive effect on NPOs’ social integration.

The nonprofit sector can be supplementary, complementary, or adversarial to a governmental agenda (Young, 2000). As the scope and scale of public services cannot be sufficiently addressed by governmental organizations alone, partnerships among multiple actors with various resources are required to tackle complex social issues and promote better public relations (Chisholm, 1992; Gray, 1989). Therefore, governmental organizations are inclined to partner with NPOs (Brinkerhoff, 2002; Huntoon, 2001; Salamon, 1999). This is particularly true for corporatist countries like Austria, where public authorities tend to outsource welfare domains to NPOs (Salamon & Anheier, 1998). Similarly, local businesses can help build connections between local communities and external parties (Ansari et al., 2012). Finally, a high density of NPOs may result in higher specialization and increased inter-organizational collaboration (Baum & Oliver, 1996). We therefore hypothesize agglomeration effects between NPOs and other organizations for inter-organizational collaboration and thus systemic integration.

#### **H1c**

A high density of organizations has a positive effect on NPOs’ systemic integration.

### Organizational Practices and their Effects on Social and Systemic Integration

The tendency of NPOs to apply business-like practices is well documented (Hersberger-Langloh et al., 2021; Hwang & Powell, 2009). For instance, NPOs hire professionals and managers to meet institutional demands (Maier et al., 2016). NPOs become more business-like across several dimensions (e.g., structurally, rhetorically, in their goal formulation) to varying degrees. Some authors refer to this phenomenon as *hybridity* (see e.g., Brandsen et al., 2005). We speak of configurations of organizational practices, considering the different instantiations of hybridity, some leaning more towards *managerialism*, some more towards *organizational democracy*.

*Managerialism* refers to mimicking the corporate model, i.e., adopting organizational forms, management knowledge, and practices (Alexander & Weiner, 1998; Horwitz, 1988; Hvenmark, 2013). Managerialism implies a shift towards market orientation, consumerism, and commodification (Maier et al., 2016). While consumerism reshapes the relation between NPOs and beneficiaries, funders and volunteers (Yngfalk & Yngfalk, 2020: 344), commodification relates to the activities and outputs (e.g. Logan & Wekerle, 2008) and echoes the Habermasian reification of social relations (Habermas, 1981: 267ff; Jütten, 2011). Additionally, we assume that organizations prefer interactions with similar organizations. Together with the reification of social relations due to managerialism, the theory of organizational homophily (Sapat et al., 2019; Spires, 2011) leads to our H2a:

#### **H2a**

Managerialism has a positive effect on NPOs’ systemic integration

In contrast, practices of *organizational democracy* require a high level of participation of members, employees and volunteers. Such participatory routines, sometimes on a grassroot level (Enjolras, 2009; LeRoux, 2009), relate with more social integration of various individual actors. Typically, business-like forms of organizing leave little room for such democratic practices based on consensus-seeking and organizational openness (Eizenberg, 2012; Maier & Meyer, 2011), workplace participation and service quality (Baines et al., 2011; Keevers et al., 2012). Due to these inherent characteristics of organizational democracy, we hypothesize:

#### **H2b**

Organizational democracy has a positive effect on NPOs’ social integration

## Data Collection and Research Approach

### Data and Research Setting

We use data from the Civic Life of Cities Lab,^1^ an international research project investigating NPOs in urban regions across the globe. For this study, we draw on survey data from the metropolitan region of Vienna, Austria, which encompasses 2.6 million inhabitants. More than 20,000 NPOs operate in this area. In accordance with the guidelines by Salamon and Sokolowski (2016), we included self-governed private organizations with a limited profit-distribution requirement and non-compulsory participation in our sample, whereas purely grant-making foundations were excluded. Data was collected from Nov 2019 to Dec 2020.^2^

We drew a random sample of 1,304 organizations from the Austrian Register of Associations, the Austrian Companies Register and a company database provided by the data provider Herolds. After removing inactive NPOs, a total of 1,117 executives of organizations (e.g. directors or presidents) were invited to participate in the survey. In total, 593 respondents completed the survey (53% response rate). Eighty percent of respondents completed the survey online, twenty percent requested a telephone or in-person interview. Respondents could choose between a German and an English questionnaire. For this study, we concentrated on the 459 NPOs located in the city of Vienna.

For neighborhood characteristics, we relied on administrative data at the level of the statistical grid unit, which was retrieved from the Austrian Central Statistics Office. The statistical grid separates the whole Austrian territory into standardized grid cells, each of which covers an area of 250 × 250 m. The city of Vienna is divided into 7,840 grid cells, which among other demographic data include per capita income, population count and the foreign population. These data were matched with information about the density of business activities, public organizations^3^ and NPOs.^4^

**Fig. 1:**
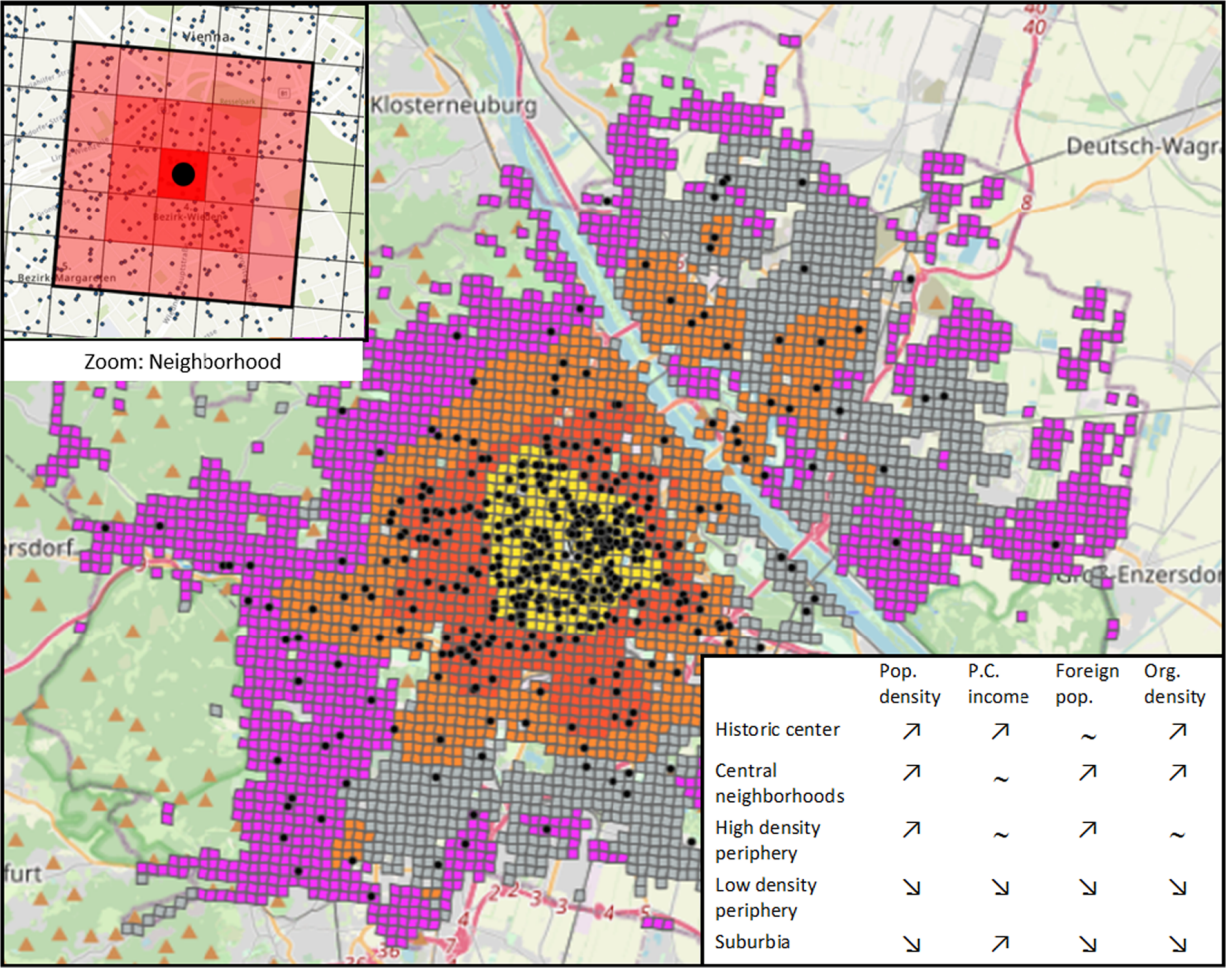
Grid cells and neighborhood types in Vienna

Figure 1 shows the city of Vienna covered by the statistical grid. Sample NPOs are indicated by black dots. The map illustrates different types of neighborhoods in Vienna in different colors, resulting from a K-means cluster analysis based on population density, per capita income, organization density, and share of immigrant population. Like many European capitals, Vienna’s neighborhood structure reveals a circular pattern: Central neighborhoods are characterized by a high population- and organization density. Income levels are highest in the historic center and the suburbs, while the share of immigrants is particularly high in the central neighborhoods around the historic core and in the densely populated peripheral areas, Vienna’s traditional working class districts. Almost half of the NPOs in our sample are located in the historic center (*n* = 204), twenty-five percent are located in the surrounding central neighborhoods (*n* = 112), and another quarter of NPOs are scattered across the three peripheral neighborhoods (high density periphery *n* = 68, low density periphery *n* = 24, and suburbia *n* = 36).

### Measures and Analysis

#### Local Neighborhood

As illustrated in the upper left corner of Fig. 1, we defined a neighborhood as the central grid cell of 250 × 250 m in which an NPO is located, plus its first order neighbors (i.e. the eight cells neighboring the NPO’s direct environment) and its second order neighbors (i.e. the 16 cells neighboring the first order neighbors). In sum, a neighborhood consists of 25 grid cells, which corresponds to a geographic area of about 1.56 km^2^.

This approach has three major advantages: First, each NPO is in the center of a relatively small neighborhood area, which is consistent with studies showing that many urban NPOs have a small radius of action (Pennerstorfer & Pennerstorfer, 2019; Wolpert, 1993). Second, the defined neighborhoods of all NPOs have exactly the same geographic size and are independent of political boundaries (e.g., districts). Third, the statistical grid units allow us to account for distance decay, which has been shown to be particularly relevant for NPOs (Bielefeld et al., 1997; Peck, 2007). We thus assume that neighborhood characteristics in the immediate vicinity of an NPO have a stronger influence on the organization than geographically more distant variables. Therefore, we discounted the first order neighbor variables by a factor of 0.33, and the second order neighbor variables by a factor of 0.66.

We included the following neighborhood-level variables: *Population density* is the total number of individuals living in a neighborhood (as control variable). *Per capita income* is measured by the average income of citizens in a neighborhood. *Share of immigrant population* is measured by the percentage of inhabitants who were not born in Austria or Germany.^5^ We then included an index for *organization density*, which is the sum of standardized values of the number of NPOs, public organizations, and business units in a grid cell.

#### Organizational Practices

The two configurations of practices, managerialism and organizational democracy, have been extracted by a factor analysis of a multitude of variables measuring practices of organizing. *Managerialism* is measured by a composite of variables indicating whether or not an NPO is characterized by management positions, a mission statement, a budget plan, a strategic plan, is externally audited, performs quantitative performance evaluation, and has recently invested in management training.

To measure *organizational democracy*, we asked NPOs about participatory practices within the organization. The following variables built the organizational democracy factor: The involvement of volunteers and members in the development of services, the appointment of management positions, and the NPO’s online-appearance. Furthermore, we asked if the organization’s constituents have the possibility to participate in meetings, view meeting minutes, and become members of the organization.

#### Social and Systemic Integration

Our measures for social and systemic integration are inspired by Brandtner and Laryea (2021:19–20). For *social integration*, we used items measuring the relevance of community building for the organization, namely building trust among citizens, promoting interaction among citizens, and creating a sense of belonging for their constituents. Respondents were asked how relevant they consider these activities for their organization: not relevant (value ‘0’), supporting tasks, side benefit (value ‘1’), and very relevant for organization (value ‘2’).

For *systemic integration*, respondents were asked about their organization’s collaboration with for-profit organizations, other NPOs, foundations, and governmental organizations. Furthermore, we included a sum index of eight items (values 0–8) measuring the involvement of NPO’s paid staff in strategic and operational decisions, a dummy variable for public events (e.g., conferences, rallies, charity events, public meetings) organized by the NPO, and a sum index (values 0–12) of four items (values 0–3) measuring the organization’s engagement in advocacy. Respondents were asked about the frequency of their organization’s involvement in policy processes. We constructed an index of social integration and one of systemic integration after performing an exploratory factor analysis.^6^

#### Controls

First, we controlled for an NPO’s size by including annual budget and member size. Second, we controlled for NPO’s geographic outreach by differentiating between locations of their primary beneficiaries: Within the district (value ‘1’), the city of Vienna (value ‘2’), Austria (value ‘3’), and beyond (value ‘4’). Third, we controlled for NPO’s primary field of activity by including three dummy variables for recreational, representative and human service organizations. Based on ICNPO categorization scheme (Salamon & Anheier, 1996: 7), we considered group 1 (culture and recreation) as recreational, groups 2, 3 and 4 (education and research, health, social services) as human services, and groups 5–12 (environment; development and housing; law, advocacy and politics; philanthropic intermediaries and voluntarism promotion; international; religion; business & professional associations, unions; not elsewhere classified) as representative. Finally, we controlled for population density of an NPO’s neighborhood. The characteristics of the independent and control variables are reported in the supplementary material. To test the effect of organizing practices and neighborhood characteristics on NPOs’ engagement in social and systemic integration, we conducted multiple linear regression analyses.

## Results

### Descriptives

Tables 2–4 in the supplementary material show descriptives, plus a few remarkable findings. With regard to systemic integration, the descriptives indicate high levels of engagement in various forms of collaboration: More than 77% of NPOs collaborate with other NPOs, 56% with public organizations, 50% with for-profit organizations, 16% with foundations, and about 43% of NPOs organize public events. Descriptives of social integration show that activities such as trust-building among people and promoting interaction amongst them are prevalent.

**Fig. 2:**
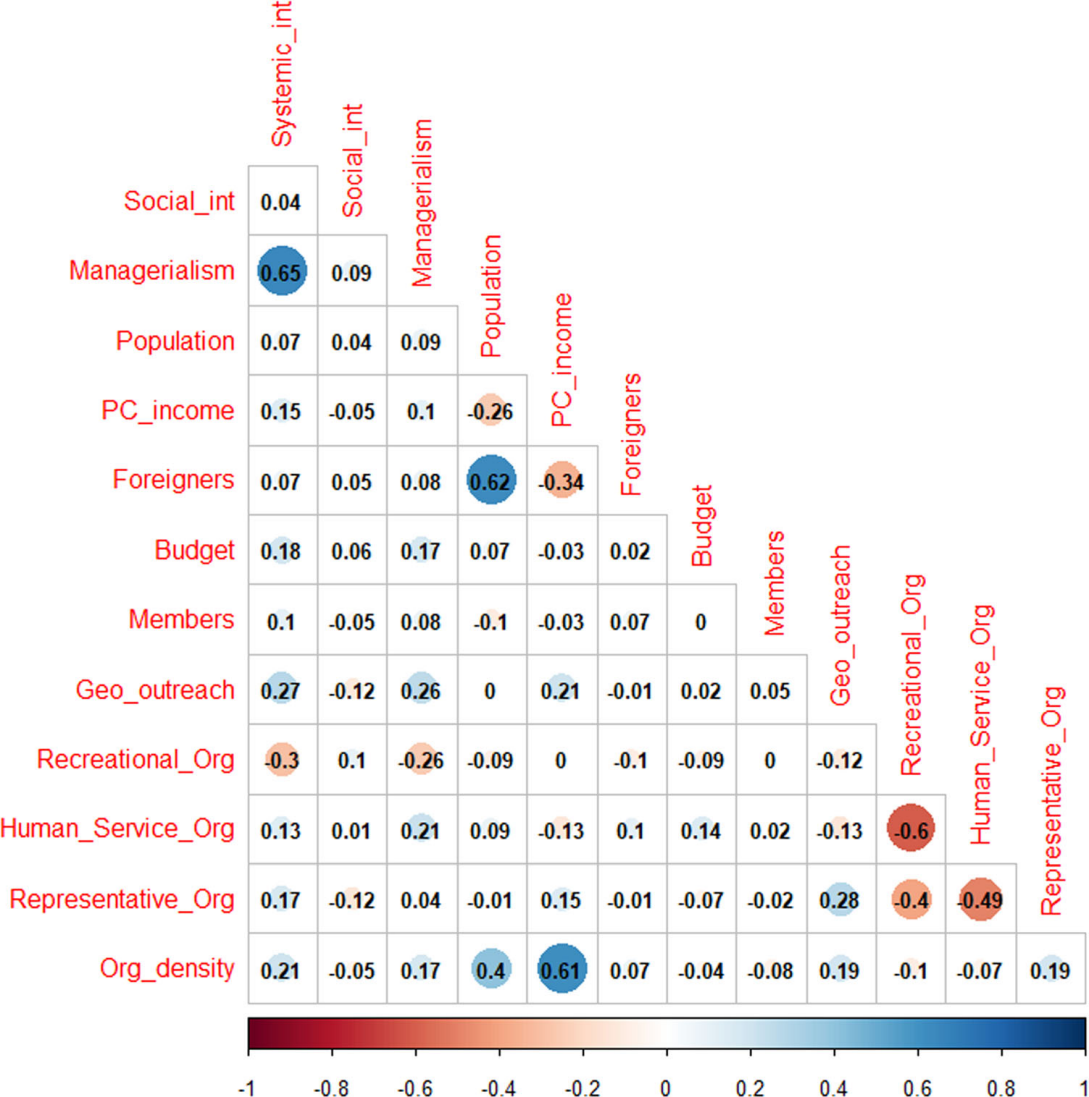
Correlation matrix

Correlations are consistent with the theoretical expectations (see Fig. 2). Within correlations, we see no support for colonialization respectively crowding out of social by systemic integration. Systemic integration strongly correlaties with managerialism (*r* = 0.65). Within neighborhood variables, organization density and per capita income (*r* = 0.61) show a high correlation. Likewise, the share of foreigners correlates with population density (*r* = 0.62). To check for multicollinearity, variance inflation factor (VIF) tests were performed. The mean VIF of both models is below 1.9, the maximum VIF is 3.6, thus not indicating multicollinearity.

### Regression Models

We conducted two regression analyses (model 1 and model 2) to test H1a-H1c and H2a-2b (Table 1). Whereas the adjusted *R*^2^ for social integration is only 0.068 (model 1), it is 0.457 for systemic integration (model 2). First, we tested whether neighborhood characteristics explain variation in social and systemic integration. H1a assumes a positive effect of low per capita income on social integration. The results, however, indicate no significant relationship, thus not confirming H1a. Similarly, neither H1b (immigrant population) and H1c (organization density) are supported by the results. Thus, all three hypotheses about the effects of neighborhood characteristics on social and systemic integration are not supported by our results.

**Table 1:** Regression results

Predictors	Model 1			Model 2		
	Social integration			Systemic integration		
	Estimates	std. Error	*p*	Estimates	std. Error	*p*
(Intercept)	− 0.22	0.58	0.706	− 0.41	0.44	0.358
Managerialism	0.17	0.05	**0.001**	0.59	0.04	**< 0.001**
Org.democracy	0.18	0.05	**< 0.001**	− 0.08	0.04	**0.035**
Scale(budget)	0.03	0.04	0.388	0.07	0.03	**0.023**
Scale(members)	− 0.05	0.04	0.230	0.04	0.03	0.145
Geo_outreach	− 0.11	0.05	**0.024**	0.06	0.04	0.103
Org_density	− 0.03	0.03	0.272	0.03	0.02	0.237
Population	0.00	0.00	0.455	− 0.00	0.00	0.411
PC_income	0.00	0.00	0.467	0.00	0.00	0.729
Foreigners	0.53	0.75	0.485	0.43	0.57	0.456
Human_service_org	-0.17	0.10	0.089	0.16	0.07	**0.033**
Representative_org	-0.29	0.11	**0.009**	0.33	0.08	**< 0.001**
Observations	435			435		
*R*^2^/*R*^2^ adjusted	0.092/0.068			0.471/0.457		

^*^p < 0.05 ** *p* < 0.01 *** *p* < 0.001

Second, we tested for effects of organizational practices on social and systemic integration. H2a predicts a positive relationship between managerialism and NPOs’ engagement in systemic integration (model 2). H2b assumes a positive relationship between organizational democracy and the NPOs’ engagement in social integration (model 1). The results of both models show positive and significant effects, thus supporting H2a and H2b.

Regarding the control variables, the results indicate that geographic outreach has a significant negative effect on social integration. Organizational size in terms of budget has a modest significant positive effect on systemic integration, while the member base has no effect in neither of the models. Finally, the results confirm our assumptions about the effects of NPOs’ primary field of activity: Compared to recreational NPOs (the reference category), NPOs operating in human services and representative organizations exhibit significantly higher levels of systemic integration. For the representative category, the results are even highly significant.

## Discussion

We analyzed the influence of neighborhood characteristics and organizational practices on NPOs’ engagement in social and systemic integration in the context of the city of Vienna. By considering how NPOs engage in both forms of integration, we advance scholarship on how NPOs create social cohesion and neighborhood vitality (e.g., Hwang & Young, 2022; Lichterman & Eliasoph, 2014; Marwell & Gullickson, 2013). We thereby add to scholarship on social capital by highlighting the organizational perspective of NPOs in enhancing urban cohesion. Particularly, we extend the understanding of the systemic aspect of urban cohesion. Our study thus provides a nuanced picture and shows that NPOs mostly engage in both forms of integration.

Concerning neighborhood effects, our findings suggest that NPOs’ engagement in social and systemic integration does not depend on neighborhood characteristics. However, at the city level, social and systemic integration vary as NPOs concentrate in central, affluent neighborhoods. As the overall density of organizations is highly correlated with the per capita income (0.61), we see an uneven distribution of overall NPOs’ integrative activities across neighborhoods. These results are consistent with previous research suggesting agglomeration effects (e.g., Bielefeld and Murdoch, 2004; Katz, 2014).

Concerning the effects of organizational practices on the different forms of integration, our study shows a positive effect of organizational democracy on social integration and a positive effect of managerialism on systemic integration. The model for social integration is weak but shows a significant influence of organizational practices; but neither neighborhood characteristics, fields of activity, nor organizational size explain how much NPOs engage in social integration. This is surprising and calls for further research.

In line with the theory of organizational homophily (Sapat et al., 2019; Spires, 2011), we find that managerialist organizations interact more with other organizations, independent of whether it concerns NPOs, businesses, and public organizations. Further, organizational size positively relates to systemic integration. Hence, NPOs concentrating on inter-organizational collaboration are characterized by managerial practices and larger budgets and tend to be located in central and more affluent neighborhoods.

Warnings of the collateral damage of managerialism in NPOs (Baines et al., 2011; Keevers et al., 2012; Maier et al., 2016) are not justified when it comes to social integration: our study shows that the managerial practices do not harm NPOs’ engagement in integration. Even organizational size has no negative influence on social integration. We thus suppose that managerial practices have pervaded NPOs, but obviously their adjustment to managerialism has mitigated colonization effects, e.g. the crowding out of social by systemic integration.

In a nutshell, our findings deliver a nuanced picture of NPOs’ engagement in integration, challenging the one-sided impression coined by prior qualitative research. There are indeed single NPOs that work in disadvantaged neighborhoods, providing neighborhood communities with networks, resources, and social capital. Yet, our study shows a lower density of NPOs in poor neighborhoods. Thus, the NPOs’ location decisions do not necessarily follow citizens’ needs. Instead, NPOs tend to concentrate in neighborhoods that are already endowed with a lively and strong organizational infrastructure.

Our study has several limitations. First, our findings are context specific. Our conclusions are bound to the particularities of a specific type of European city. Hence, studies should replicate the research design in other urban contexts to scrutinize the local embeddedness of NPOs and the differences in their engagement in social and systemic integration.

Second, due to the cross-sectional character of our data, we can show relations between organizational practices and forms of integration but no causality in a strict sense. Managerialism forwards social and systemic integration, and organizational democracy forwards social integration, yet the reverse causality is plausible too. Furthermore, our low *R*^2^ for the social integration model demands caution, whereas the results for systemic integration allow us to paint a much sharper picture.

Third, our measures of social integration aim at NPOs’ goals, whereas our measures of systemic integration aim at NPOs’ actual activities, providing a further argument why our results for systemic integration are stronger. Fourth, our location data only covers NPOs’ headquarters. For larger NPOs with many branches, this might still yield a bias towards inner-city locations. It is remarkable that smaller grassroot-NPOs located in the outskirts and poorer neighborhoods do not compensate for this bias. However, as social integration negatively relates with geographical outreach, there is no direct evidence that branches and subsidiaries of inner-city NPOs compensate for this structural deficit.

Finally, there are implications for urban planning and policymaking. Our results suggest strong agglomeration effects that aggravate differences in service provision and infrastructure even in an affluent city like Vienna with low social segregation (Kohlbacher & Reeger, 2020; Ranci & Sabatinelli, 2020). Assuming that NPOs deliver relevant contributions to urban cohesion, urban planning should consider the impact of placing public organizations like schools, hospitals, and kindergartens. The same applies for the public support of local business activities. Similarly, the location of NPOs matters. Offering affordable office space for NPOs in public premises or subsidizing rents for NPOs in vulnerable urban neighborhoods could contribute to a more effective spatial outreach of civil society and thus foster urban cohesion.

## Supplementary Information

Below is the link to the electronic supplementary material.

Supplementary file1 (DOCX 27 KB)

## Footnotes

1 https://pacscenter.stanford.edu/research/civic-life-of-cities-lab/

2 We are aware that NPOs are greatly affected by the Covid-19 pandemic and relating social distancing measures, with possible effects onon integration activities. To rule out possible effects of the pandemic on the data collection, we split the sample in two groups—respondents who have answered the questionnaire before March 2020 and during the pandemic—which did not yield significant differences.

3 both provided by the Austrian Chamber of Commerce.

4 retrieved from the Austrian Register of Associations.

5 Germans are the third largest immigrant group in Vienna. However, due to the close cultural proximity and the common language, we did not treat them as immigrants.

6 The survey questions, the characteristics of the dependent variables and the results of the factor analysis are reported in the supplementary material.
