# Supplementary material for: Nonprofits for Cohesive Cities: Neighborhood Characteristics, Organizational Practices, and their Effects on Social and Systemic Integration
Source: Voluntas. 2023 May 5:1–12. Online ahead of print. doi: 10.1007/s11266-023-00571-1 (PMC10161977; doi:10.1007/s11266-023-00571-1)
Supplement: Supplementary file 1 — Supplementary file1 (DOCX 27 KB) [file 11266_2023_571_MOESM1_ESM.docx]

**Supplementary Material**

**Table 2: Descriptives and factor loadings of the dependent variables**

|  | **N** | **Mean** | **Min** | **Max** |  | **Factor Loadings** | |
| --- | --- | --- | --- | --- | --- | --- | --- |
| **Systemic Integration** | **459** | **0.16** | **-1.37** | **1.94** |  | **Systemic Integration** | **Social Integration** |
| Collaboration with for-profits | 593 | 0.49 | 0 | 1 |  | 0.524 |  |
| Collaboration with nonprofits | 593 | 0.77 | 0 | 1 |  | 0.421 |  |
| Collaboration with foundations | 593 | 0.16 | 0 | 1 |  | 0.374 |  |
| Collaboration with government | 593 | 0.56 | 0 | 1 |  | 0.635 |  |
| Involvement of staff | 593 | 2.12 | 0 | 8 |  | 0.656 |  |
| Public events organized | 593 | 0.43 | 0 | 1 |  | 0.527 |  |
| Engagement in advocacy | 593 | 10.60 | 0 | 12 |  | 0.437 |  |
| **Social Integration** | **459** | **-0.02** | **-2.43** | **0.88** |  |  |  |
| Building trust | 593 | 1.48 | 0 | 2 |  |  | 0.621 |
| Promoting interaction | 593 | 1.52 | 0 | 2 |  |  | 0.739 |
| Creating sense of belonging | 593 | 1.45 | 0 | 2 |  |  | 0.670 |

**Table 3: Descriptives and factor loadings of the organizing practices variables**

|  | **N** | **Mean** | **Min** | **Max** |  | **Factor Loadings** | |
| --- | --- | --- | --- | --- | --- | --- | --- |
| **Managerialism** | **459** | 0.14 | -1.74 | 1.35 |  | **Managerialism** | **Org. Democracy** |
| Management positions | 593 | 0.47 | 0 | 1 |  | 0.448 |  |
| Mission statement | 593 | 0.56 | 0 | 1 |  | 0.560 |  |
| Budget plan | 593 | 0.70 | 0 | 1 |  | 0.638 |  |
| Strategic plan | 593 | 0.60 | 0 | 1 |  | 0.622 |  |
| External audit | 593 | 0.54 | 0 | 1 |  | 0.623 | -0.189 |
| Quant. evaluation | 593 | 0.67 | 0 | 1 |  | 0.464 |  |
| Management training | 593 | 0.28 | 0 | 1 |  | 0.353 |  |
| **Organizational democracy** | **459** | -0.05 | -1.11 | 1.84 |  |  |  |
| Involve volunt. & members: services | 593 | 0.44 | 0 | 1 |  |  | 0.429 |
| Involve volunt. & members: appoint. mgmt | 593 | 0.29 | 0 | 1 |  | -0.199 | 0.431 |
| Involve volunt. & members: online app. | 593 | 0.35 | 0 | 1 |  |  | 0.381 |
| Constituents participate meetings | 593 | 0.39 | 0 | 1 |  |  | 0.651 |
| Constituents view meeting minutes | 593 | 0.26 | 0 | 1 |  |  | 0.590 |
| Constituents become members | 593 | 0.50 | 0 | 1 |  |  | 0.570 |

**Table 4: Descriptives of the neighborhood variables and control variables**

| **Neighborhood Characteristics** | N | Mean | | Min | | Max |
| --- | --- | --- | --- | --- | --- | --- |
| NPO-density (scale) | 444 | | 30.66 (0) | | 1.33 (-2) | 69.67 (2) |
| Business activities (scale) | 444 | | 1781.08 (0) | | 10.33 (-1) | 5,602.33 (3) |
| Public organization density (scale) | ~~444~~ | | 124.08 (0) | | 0.67 (-1) | 446.00 (4) |
| Organization density | 444 | | 0 | | -4 | 5 |
| Population density | 444 | | 10,457.07 | | 144.00 | 22,395.00 |
| PC Income in € / month | 444 | | 1,502.89 | | 682.82 | 2,141.34 |
| % Foreign. | 444 | | 0.33 | | 0.09 | 0.55 |
| **Control Variables** |  | | | | | |
| Budget | 457 | | 7,145,337 | | 0 | 800,000,000 |
| Members | 450 | | 10,311.39 | | 0 | 2,300,000 |
| Geographic Outreach | 459 | | 2.43 | | 1 | 4 |
| Recreational Org | 459 | | 0.33 | | 0 | 1 |
| Representative Org | 459 | | 0.25 | | 0 | 1 |
| Human Service Org | 459 | | 0.43 | | 0 | 1 |

**List of survey items**

**Systemic Integration**

**Does your organization collaborate with any of the following organizations for any of the following purposes?**

|  | with for-profit businesses | with government agencies/public organizations | with other nonprofit organizations | with foundations |
| --- | --- | --- | --- | --- |
| Delivering services for our target group | ⬜ | ⬜ | ⬜ | ⬜ |
| Advocacy | ⬜ | ⬜ | ⬜ | ⬜ |
| Building community between people | ⬜ | ⬜ | ⬜ | ⬜ |
| Building the capacities of our organization | ⬜ | ⬜ | ⬜ | ⬜ |
| Commercial purposes | ⬜ | ⬜ | ⬜ | ⬜ |
| Recruiting volunteers | ⬜ | ⬜ | ⬜ | ⬜ |
| Organizing events | ⬜ | ⬜ | ⬜ | ⬜ |

**Who is routinely involved in the following tasks?**

|  | top leader | board | paid staff | volunteers | members | people from the target group | external consultants | others |
| --- | --- | --- | --- | --- | --- | --- | --- | --- |
| Developing the strategic plan | ⬜ | ⬜ | ⬜ | ⬜ | ⬜ | ⬜ | ⬜ | ⬜ |
| Develop ideas for new programs/services to be offered by the organization | ⬜ | ⬜ | ⬜ | ⬜ | ⬜ | ⬜ | ⬜ | ⬜ |
| Selecting people for leadership positions | ⬜ | ⬜ | ⬜ | ⬜ | ⬜ | ⬜ | ⬜ | ⬜ |
| Creating publicly available report about the organization’s activities | ⬜ | ⬜ | ⬜ | ⬜ | ⬜ | ⬜ | ⬜ | ⬜ |
| Planning the budget | ⬜ | ⬜ | ⬜ | ⬜ | ⬜ | ⬜ | ⬜ | ⬜ |
| Developing the mission statement | ⬜ | ⬜ | ⬜ | ⬜ | ⬜ | ⬜ | ⬜ | ⬜ |
| Contributing content to social media | ⬜ | ⬜ | ⬜ | ⬜ | ⬜ | ⬜ | ⬜ | ⬜ |
| Contributing content to website | ⬜ | ⬜ | ⬜ | ⬜ | ⬜ | ⬜ | ⬜ | ⬜ |

**Approximately how many events did your organization host or sponsor last year?**

|  | number of events |
| --- | --- |
| Recreational activities |  |
| Charity events or fundraisers |  |
| Festivals or celebrations |  |
| Conferences, lectures, panel discussions, seminars |  |
| Public meetings, hearings, petitions |  |
| Rallies, demonstrations, marches |  |
| Volunteer work days (e.g. cleaning days, visiting days) |  |
| Others: |  |

**Has your organization been involved in policy-making processes over the last three years?**

|  | routinely involved | occasionally involved | never involved |
| --- | --- | --- | --- |
| At the local level (e.g. in a Viennese district, in a municipality in Lower Austria) | O | O | O |
| At the provincial level (e.g. City of Vienna, province of Lower Austria) | O | O | O |
| At the federal level in Austria | O | O | O |
| At the European level* | O | O | O |
| At the global level* | O | O | O |

* were combined to form "at the international level".

**Social integration**

**How important are the following activities to the mission of your organization?**

|  | critical to mission | supports mission/desired side-effect | unimportant |
| --- | --- | --- | --- |
| Building trust between people | O | O | O |
| Promoting regular interactions between people | O | O | O |
| Providing a place for people to feel a sense of belonging | O | O | O |

**Managerialism**

**Over the past 3 years, has your organization had or changed positions responsible for any of the following tasks?**

|  | already existed | created now | expanded existing | contracted |
| --- | --- | --- | --- | --- |
| Other managerial tasks | ⬜ | ⬜ | ⬜ | ⬜ |

|  | Yes | No, not anymore | No, we never had |
| --- | --- | --- | --- |
| Mission statement | O | O | O |
| Strategic plan (=a document about what your organization wants to achieve, and how it will do so) | O | O | O |
| Written budget plan | O | O | O |
| Financial audit by external professional auditor | O | O | O |

**Does your organization have any of the following?**

**How does your organization monitor or evaluate whether it is successful?**

⬜ Input metrics (e.g., how many working hours or how much money went into a particular offer by the organization)

⬜ Output metrics (e.g., how many people made use of a particular offer by the organization)

⬜ Impact metrics (e.g., whether a particular offer by the organization had a positive effect on the users’ behavior)

**Have you or volunteers/staff members participated in training programs on any of the following topics as part of their work for the organization?**

|  | You | Volunteers/staff members |
| --- | --- | --- |
| Other management topics | ⬜ | ⬜ |

**Organizational democracy**

**Who is routinely involved in the following tasks?**

|  | Top leader | Board | Paid staff | Volunteers | Members | People from the target group | External consultants | Others |
| --- | --- | --- | --- | --- | --- | --- | --- | --- |
| Develop ideas for new programs/services to be offered by the organization | ⬜ | ⬜ | ⬜ | ⬜ | ⬜ | ⬜ | ⬜ | ⬜ |
| Selecting people for leadership positions | ⬜ | ⬜ | ⬜ | ⬜ | ⬜ | ⬜ | ⬜ | ⬜ |
| Contributing content to social media* | ⬜ | ⬜ | ⬜ | ⬜ | ⬜ | ⬜ | ⬜ | ⬜ |
| Contributing content to website* | ⬜ | ⬜ | ⬜ | ⬜ | ⬜ | ⬜ | ⬜ | ⬜ |

*were combined to form “contribute to online appearance”

**What opportunities do people from your target group have to participate in the organization’s decisions?**

⬜ None

⬜ Formally join the organization (e.g. become a member) to have more participation rights

⬜ Participate in public meetings of committees or board

⬜ Access meeting minutes

⬜ Visit us in person and give us feedback

⬜ Comment on documents and reports

⬜ Other opportunities
